# What is the pipeline for future medications for obesity?

**DOI:** 10.1038/s41366-024-01473-y

**Published:** 2024-02-01

**Authors:** Eka Melson, Uzma Ashraf, Dimitris Papamargaritis, Melanie J. Davies

**Affiliations:** 1 Diabetes Research Centre, University of Leicester College of Life Sciences, Leicester, UK; 2 Leicester Diabetes Centre, Leicester General Hospital, Leicester, LE5 4PW UK; 3 Department of Diabetes and Endocrinology, Kettering General Hospital NHS Foundation Trust, Kettering, NN16 8UZ UK

**Keywords:** Obesity, Obesity

## Abstract

Obesity is a chronic disease associated with increased risk of obesity-related complications and mortality. Our better understanding of the weight regulation mechanisms and the role of gut-brain axis on appetite has led to the development of safe and effective entero-pancreatic hormone-based treatments for obesity such as glucagon-like peptide-1 (GLP-1) receptor agonists (RA). Semaglutide 2.4 mg once weekly, a subcutaneously administered GLP-1 RA approved for obesity treatment in 2021, results in 15–17% mean weight loss (WL) with evidence of cardioprotection. Oral GLP-1 RA are also under development and early data shows similar WL efficacy to semaglutide 2.4 mg. Looking to the next generation of obesity treatments, combinations of GLP-1 with other entero-pancreatic hormones with complementary actions and/or synergistic potential (such as glucose-dependent insulinotropic polypeptide (GIP), glucagon, and amylin) are under investigation to enhance the WL and cardiometabolic benefits of GLP-1 RA. Tirzepatide, a dual GLP-1/GIP receptor agonist has been approved for glycaemic control in type 2 diabetes as well as for obesity management leading in up to 22.5% WL in phase 3 obesity trials. Other combinations of entero-pancreatic hormones including cagrisema (GLP-1/amylin RA) and the triple agonist retatrutide (GLP-1/GIP/glucagon RA) have also progressed to phase 3 trials as obesity treatments and early data suggests that may lead to even greater WL than tirzepatide. Additionally, agents with different mechanisms of action to entero-pancreatic hormones (e.g. bimagrumab) may improve the body composition during WL and are in early phase clinical trials. We are in a new era for obesity pharmacotherapy where combinations of entero-pancreatic hormones approach the WL achieved with bariatric surgery. In this review, we present the efficacy and safety data for the pipeline of obesity pharmacotherapies with a focus on entero-pancreatic hormone-based treatments and we consider the clinical implications and challenges that the new era in obesity management may bring.

## Introduction

Obesity is a chronic disease characterised by excess adiposity that impairs health and affects about 650 million people worldwide [1, 2]. It increases the risk for multiple metabolic complications including type 2 diabetes (T2D), metabolic-dysfunction associated steatotic liver disease (MASLD, previously known as non-alcoholic fatty liver disease), cardiovascular disease as well as many mechanical complications such as osteoarthritis and obstructive sleep apnoea (OSA) [3].

Lifestyle interventions (including diet, exercise and behavioural changes) are the cornerstone of obesity management with diverse benefits, but even the most intensive lifestyle interventions result in up to 10% mean weight loss (WL) and weight maintenance remains a challenge, as 80% of WL is expected to be regained over the next 5 years [4, 5]. The main drivers for weight regain after significant WL include the persistence of a lower resting metabolic rate and the increased appetite, possibly mediated through long lasting increased orexigenic and decreased anorexigenic signals [2, 6]. Despite that 5–10% WL is clinically beneficial, greater WL may be required for the individual to improve or achieve remission of some obesity-related complications [7, 8]. Bariatric surgery can result in 25–30% mean WL and long-term weight maintenance, however, it is not scalable at the population level and people may be hesitant to this option due to the perceived risk of postoperative complications [9, 10].

**Fig. 1:**
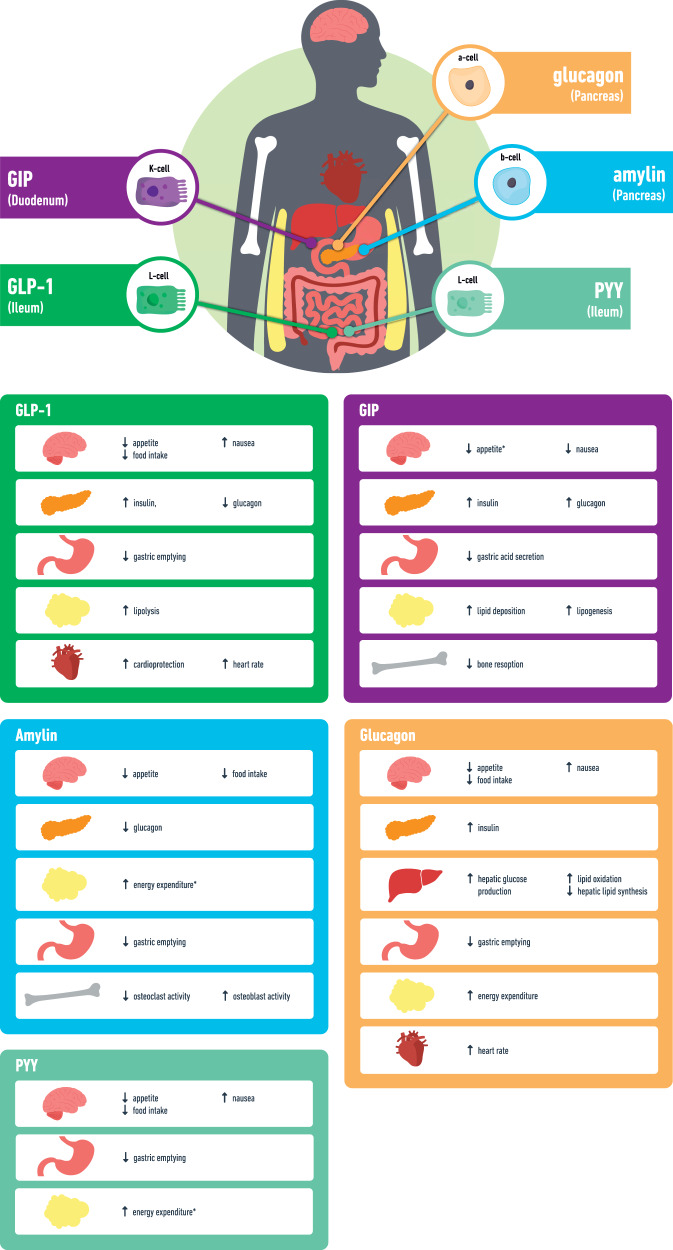
Secretion and main actions of the gut hormones used in the pipeline obesity treatments. GLP-1 glucagon like peptide-1, GIP glucose-dependent insulinotropic polypeptide, PYY peptide YY, *data mainly from animal studies.

Understanding better the role of the entero-pancreatic hormones in the regulation of feeding, appetite and glycaemia (Fig. 1) has led to the development of the glucagon-like peptide-1 (GLP-1) receptor agonists (RA) as safe and effective treatments for T2D and obesity. Semaglutide 2.4 mg once weekly is the latest approved GLP-1 RA for obesity management (2021) and results in 15–17% mean WL through appetite reduction [11, 12].

However, there is still a significant difference between the WL that can be achieved with bariatric surgery and the currently approved obesity pharmacotherapies and there is heterogeneity in treatment responses with GLP-1 RA (for example people with T2D may achieve less WL compared to those without diabetes in clinical trials, despite similar lifestyle interventions) [13, 14]. Additionally, the currently approved GLP-1 RA for obesity treatment are in injectable form and some people may be reluctant to consider injectable treatments [15].

**Fig. 2:**
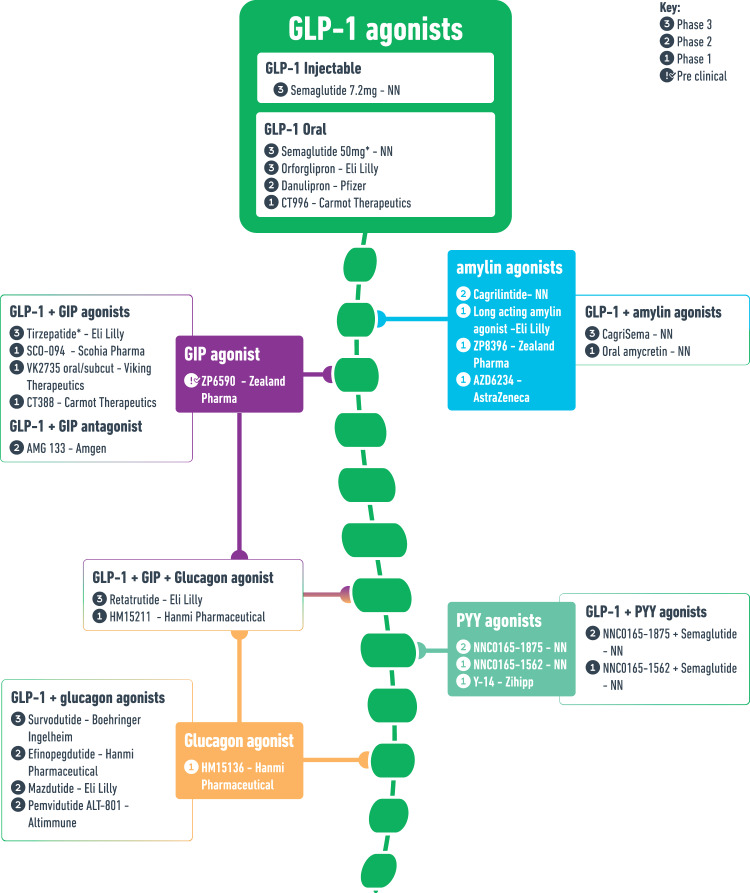
Glucagon-like peptide-1 as the backbone of the pipeline for gut hormone-based obesity treatments. GLP-1 glucagon like peptide-1, GIP glucose-dependent insulinotropic polypeptide, PYY peptide YY, NN: novo nordisk, *completed phase 3 trials for obesity.

Oral GLP-1 RA are under development to improve convenience, acceptance, and adherence and may provide an additional option to support obesity management. Additionally, a large pipeline of entero-pancreatic hormone-based pharmacotherapies is under development, with the aim to enhance and/or compliment the efficacy and mechanisms of action of GLP-1 RA (Table 1, Fig. 2). Tirzepatide is the first combination of entero-pancreatic hormones [dual GLP-1 and glucose-dependent insulinotropic polypeptide (GIP) RA] that has been approved for T2D management based on the findings from the phase 3 SURPASS programme. The marked WL achieved with tirzepatide in the SURPASS trials led to the phase 3 SURMOUNT programme, assessing tirzepatide as treatment for obesity [16]. Other non-entero-pancreatic hormone-based pharmacotherapies such as bimagrumab and growth differentiation factor 15 (GDF-15) are also under investigation.

**Table 1:** Pipeline for future obesity medications.

Name	Dose	Administration	Mechanism of action	Company	Expected completion date	Clinical Trials gov	Other indication(s)
**Phase 3 obesity trials**							
Semaglutide*	50 mg	PO, OD	GLP-1 RA	Novo Nordisk	Completed	NCT05035095	Phase 3 - T2D
Orforglipron	NA	PO, OD	GLP-1 RA	Eli Lilly	September-2027	NCT05869903	Phase 3 - T2D, CV outcomes in T2D
Semaglutide	7.2 mg	SC, OW	GLP-1 RA	Novo Nordisk	NA	NA	NA
Tirzepatide*	5–15 mg	SC, OW	GLP-1 RA + GIP RA	Eli Lilly	Completed	NCT04184622	Phase 3 - T2D, HFpEF, OSA, CV outcomes in T2D, morbidity and mortality in obesity Phase 2 - MASH, CKD
CagriSema	2.4 mg/2.4 mg	SC, OW	GLP-1 RA + Amylin RA	Novo Nordisk	October-2026	NCT05567796	Phase 3 - T2D, CV outcomes
Survodutide	3.6–6 mg	SC, OW	GLP-1 RA + GCG RA	Boehringer Ingelheim	Completed	NCT04667377	Phase 2 - T2D, MASH
Mazdutide	4–6 mg	SC, OW	GLP-1 RA + GCG RA	Innovent Biologics	April-2024	NCT05607680	Phase 3 - T2D Phase 1 CKD
Mazdutide	9 mg	SC, OW	GLP-1 RA + GCG RA	Innovent Biologics	September 2025	NCT06164873	NA
Retatrutide	4–12 mg	SC, OW	GLP-1 RA + GIP RA + GCG RA	Eli Lilly	May-2026	NCT05929066	Phase 3 - T2D, OA Phase 2 - CKD
**Phase 2 obesity trials**							
Danuglipron	40–200 mg	PO, BD	GLP-1 RA	Pfizer	Completed	NCT04707313	NA
Cagrilintide	0.3–4.5 mg	SC, OW	Amylin RA	Novo Nordisk	Completed	NCT03856047	Phase 1 - MASH
PYY 1875	0.03–2.4 mg	SC, NA	PYY RA	Novo Nordisk	Completed	NCT03707990	NA
Efinopegdutide	5–10 mg	SC, OW	GLP-1 RA + GCG RA	Hanmi Pharmaceutical	Completed	NCT03486392	Phase 2 - T2D, MASH, MASLD
Pemvidutide	1.2–2.4 mg	SC, OW	GLP-1 RA + GCG RA	Altimmune	Completed	NCT05295875	Phase 2 - MASH, MASLD Phase 1 – T2D
AMG 133	NA	SC, once monthly	GLP-1 RA + GIP receptor antagonist	Amgen	January-2025	NCT05669599	NA
NNC0165-1875 + Semaglutide	1–2 mg + 2.4 mg	SC, every 2 to 4 weeks	GLP-1 RA + PYY RA	Novo Nordisk	Completed	NCT04969939	NA
Dapiglutide	4–6 mg	SC, OW	GLP-1 RA + GLP2 RA	Zealand Pharma	August-2024	NCT05788601	NA
Bimagrumab + Semaglutide	30 mg/kg + 1–2.4 mg	IV, every 4 weeks (Bimagrumab) + SC, OW	Activin receptor II inhibition + GLP-1 RA	Versanis Bio	September-2025	NCT05616013	NA
S-309309	NA	PO, OD	MGAT2	Shionogi	May-2024	NCT05925114	NA
**Phase 1 obesity trials**							
CT-996	NA	PO, OD	GLP-1 RA	Carmot Therapeutics	November-2024	NCT05814107	NA
Long-acting amylin agonist	NA	NA	Amylin RA	Eli Lilly	NA	NA	NA
AZD6234	NA	SC, OW	Amylin RA	AstraZeneca	December-2023	NCT05511025	NA
ZP8396	NA	SC, OW	Amylin RA	Zealand Pharma	May-2024	NCT05613387	NA
HM15136	NA	SC, frequency not stated	Glucagon RA	Hanmi Pharmaceutical	Completed	NCT04032782	NA
NNC0165-1562	NA	SC, OW	PYY RA	Novo Nordisk	Completed	NCT02568306	NA
Y-14	9-36mg	SC, OW/every 2 weeks	PYY RA	Zihipp	Completed	NCT0367311	NA
VK2735	NA	PO, frequency not stated	GLP-1 RA + GIP RA	Viking Therapeutics	NA	NA	Phase 1 – MASH
VK2735	NA	SC, OW	GLP-1 RA + GIP RA	Viking Therapeutics	December-2023	NCT05203237	Phase 1 – MASH
SCO-094	NA	PO, frequency not stated	GLP-1 RA + GIP RA	Scohia Pharma	NA	NA	Phase 1 - T2D, MASH
CT-388	5–12 mg	SC, OW	GLP-1 RA + GIP RA	Carmot Therapeutics	Completed	NCT04838405	Phase 1 - T2D
Amycretin (NNC0487-0111)	1–100 mg	PO, OD	GLP-1 RA + Amylin RA	Novo Nordisk	November-2024	NCT05369390	NA
Dacra QW II	NA	NA	Amylin RA + calcitonin RA	Eli Lilly	NA	NA	NA
NNC0165-1562 and Semaglutide	NA	SC, OW	PYY RA + GLP-1RA	Novo Nordisk	Completed	NCT03574584	NA
HM15211	NA	SC, OW	GLP-1 RA + GIP RA + GCG RA	Hanmi Pharmaceutical	Completed	NCT03374241	Phase 2 – MASH
NNC0247-0829	NA	SC, OW	GDF15 analogue	Novo Nordisk	Completed	NCT04010786	NA
JNJ-9090/CIN-109	NA	SC, OW/Twice weekly	GDF15 analogue	CinRx Pharma	NA	NA	NA
SCO-267	NA	PO, OD	G-protein-coupled receptor 40	Scohia Pharma	Completed	JapicCTI-195057	Phase 1 - MASH
**Preclinical status**							
ZP6590	NA	NA	GIP RA	Zealand Pharma	NA	NA	NA

*Completed phase 3 trials for obesity

*T2D* type 2 diabetes, *HFpEF* heart failure with preserved ejection fraction, *MASH* metabolic dysfunction-associated steatohepatitis, *MASLD* metabolic dysfunction-associated steatotic liver disease, *CKD* chronic kidney disease, *CV* cardiovascular, *OA* osteoarthritis, *OSA* obstructive sleep apnoea, *RA* receptor agonist, *GLP-1* glucagon-like peptide-1, *GIP* glucose-dependent insulinotropic polypeptide, *GCG* glucagon, *PYY* peptide YY, *GDF15* Growth/differentiation factor-15, *MGAT2* Monoacylglyceroltransferase 2, *SC* subcutaneous, *PO* oral, *IV* intravenous, *OD* once-daily, *BD* twice daily, *OW* once-weekly, *NA* data not available.

In this review, we discuss the pipeline of obesity pharmacotherapies with focus on entero-pancreatic hormone-based molecules, and we evaluate the data from early and late phase clinical trials on their safety and efficacy on WL in people with and without T2D. We also consider the effect of these molecules on other obesity-related complications [glycaemia in people with T2D and on liver fat content in people with MASLD/metabolic dysfunction-associated steatohepatitis (MASH, previously known as non-alcoholic steatohepatitis)] and we discuss the potential clinical implications and challenges that the new era in obesity pharmacotherapy will bring.

## Pipeline of obesity pharmacotherapies

### GLP-1 receptor agonists

GLP-1 RA increase satiety, reduce food intake and delay gastric emptying whilst they also stimulate insulin release and inhibit glucagon secretion in a glucose-dependent manner (Fig. 1) [17, 18]. Subcutaneous liraglutide 3 mg (once daily) and semaglutide 2.4 mg (once weekly) have been approved for obesity management and a higher dose of subcutaneous semaglutide (7.2 mg once weekly) is currently assessed in a phase 3 trial (Table 1). However, people may be reluctant to consider injectable treatments. To overcome the barriers related to injections, semaglutide has become available in oral form containing an absorption enhancer which facilitates uptake through gastric mucosa [19]. Oral semaglutide needs to be taken at the morning with 120 mls water, 30 min before any meal intake in order to ensure adequate absorption. Based on the PIONEER programme, oral semaglutide has been licensed for people with T2D and the 14 mg dose, leads to HbA1c improvement up to −1.4% and WL up to 4.4 kg [20].

### Oral semaglutide 50 mg

**Fig. 3:**
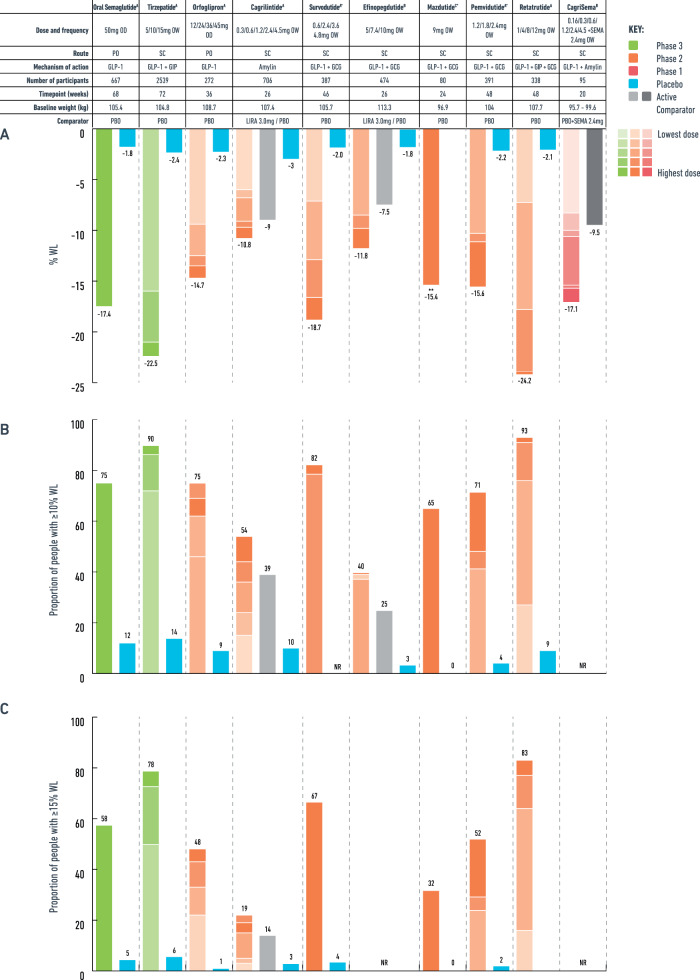
Weight loss with the obesity pharmacotherapies pipeline in people without diabetes. **A** Mean % weight loss, **B** Proportion of people achieving ≥10% weight loss, **C** Proportion of people achieving ≥15% weight loss. OD once daily, OW once weekly, PO oral, SC subcutaneous, GLP-1 glucagon like peptide-1, GIP glucose-dependent insulinotropic polypeptide, GCG glucagon, PBO placebo, LIRA liraglutide, SEMA semaglutide, WL weight loss, NR not reported or not available. ^A^for efficacy estimand data, ^B^main analysis presented, as efficacy estimand not available, ^C^unclear whether efficacy estimand or treatment-regimen estimand, *data from published abstract, presentation, clinicaltrial.gov or from press-release by the manufacturing company, **estimated treatment difference.

In people with obesity without T2D, a 68-week phase 3 trial (OASIS-1) assessed the safety and efficacy of oral semaglutide 50 mg once daily vs. placebo when combined with a moderate-intensity lifestyle intervention (Supplementary Table 1). Oral semaglutide 50 mg resulted in 17.4% WL compared to 1.8% with placebo (Fig. 3), with improvements in multiple cardiometabolic risk factors (Table 2) [21].

**Table 2:** Efficacy and safety outcomes with the obesity medications pipeline for people without diabetes.

Medication	Semaglutide [21] 50 mg^A^	Tirzepatide [46] 5, 10, 15 mg^A^	Orforglipron [25] 12, 24, 36, 45 mg^A^	Cagrilintide [87] 0.3, 0.6, 1.2, 2.4, 4.5 mg^A^	Survodutide* [68] 0.6, 2.4, 3.6, 4.8 mg^A,B^	Efinopegdutide [77] 5, 7.4, 10 mg^B^	Mazdutide [70] 3, 4.5, 6.0 mg^B^	Mazdutide* [71] 9 mg^C^	Pemvidutide (ALT-801)* [74] 1.2, 1.8, 2.4 mg^A^	Retatrutide [81] 1, 4, 8, 12 mg^A^	CagriSema [76] (0.16, 0.3, 0.6, 1.2, 2.4, 4.5 mg/2.4 mg)^B^
**Route and frequency**	PO, OD	SC, OW	PO, OD	SC, OW	SC, OW	SC, OW	SC, OW	SC, OW	SC, OW	SC, OW	SC, OW
**Comparator**	vs. plb	vs. plb	vs. plb	vs. (i) lira 3 mg (ii) plb	vs. plb	vs. (i) lira 3 mg vs. (ii) plb	vs. plb	vs. plb	vs. plb	vs. plb	vs. plb + sema 2.4 mg
**MoA**	GLP-1	GLP-1 + GIP	GLP-1	Amylin	GLP-1 + GCG	GLP-1 + GCG	GLP-1 + GCG	GLP-1 + GCG	GLP-1+ GCG	GLP-1+ GIP + GCG	GLP-1 + amylin
**Trial phase**	3	3	2	2	2	2	2	2	2	2	1
**Trial duration (weeks)**	68	72	36	26	46	26	24	24	48	48	20
**Participants**	334 vs. 333	1896 vs. 643	282 vs. 50	506 vs. (i) 99 (ii) 101	308 vs. 77	295 vs. (i) 119 (ii) 60	186 vs. 62	60 vs. 20	294 vs. 97	268 vs. 70	71 vs. 24
**Efficacy outcomes**											
**WL (%)**	−17.4% vs. 1.8%	−16% to −22.5% vs. −2.4%	−9.4% to 14.7% vs. −2.3%	−6% to −10.8% vs. (i) −9% (ii)−3%	−6.8% to −18.7% vs. −2%^A^	−8.5% to −11.8% vs. (i) −7.5% (ii) −1.8%	−6.7% to −11.3% vs. +1.0%	−15.4% (ETD)	−10.3% to −15.6% vs. −2.2%	−8.7% to −24.2% vs. −2.1%	−8.3% to −17.1% vs. −9.5%
**≥5% WL**	89% vs. 24%	89–96% vs. 28%	72–92% vs. 24%	58–89% vs. (i) 76% (ii) 31%	83% with 4.8 mg vs. 26%^B^	54–80% vs. (i) 51% (ii) 13%	58–80% vs. 4.8%	82% vs. 0%	69–84% vs. 25%	64–100% vs. 27%	NA
**≥10% WL**	75% vs. 12%	73–90% vs. 14%	46–75% vs. 9%	15–54% vs. (i) 39% (ii) 10%	69% with 4.8 mg vs. 11%^B^	37–40% vs. (i) 25% (ii) 3%	19–51% vs. 0%	65% vs. 0%	43–71% vs. 4%	27–93% vs. 9%	NA
**≥15% WL**	58% vs. 5%	50–78% vs. 6%	22–48% vs. 1%	3–19% vs. (i) 14% (ii) 3%	55% with 4.8 mg vs. 6%^B^	NA	10–25% vs. 0%	32% vs. 0%	21–52% vs. 2%	16–83% vs. 2%	NA
**≥20% WL**	37% vs. 2%	32–63% vs. 1%	NA	NA	33% with 4.8 mg vs. 0%^A^	NA	NA	22% vs. 0%	10–32% vs. 2%	6–63% vs. 1%	NA
**WC change (cm)**	−15.2 vs. −2.6	−14.6 to −19.9 vs. −3.4	−9.6 to −13.6 vs. −4.0	−5.8 to −9.2 vs. (i) 7.8 (ii) −4.4	−8.0 to −16.0 vs. −4.0^B^	NA	−5.6 to −8.8 vs. −1.1	NA	NA	−6.5 to −19.6 vs. −2.6	NA
**SBP change (mmHg)**	−8.4 vs. −0.8	−7.0 to −8.2 vs. −1.2	−6.9 to −12.1 vs. −2.2	−4.7 to −8 vs. (i) −4.3 (ii) −3.6	−6.2 to −8.7 vs. −2.5^B^	NA	−1.3 to −6.9 vs. +2.9	NA	−1.6 to −4.6 vs. +3.5	−3 to −12.1 vs. −2.3	NA
**DBP change (mmHg)**	−3.0 vs. −1.0	−4.6 to −5.5 vs. −1.0	−1.3 to −3.0 vs. −3.1	−2.2 to −5.7 vs. (i) −1.6 (ii) −2.1	−3.3 to −4.8 vs. −1.9	NA	−0.6 to −3.6 vs. +0.9	NA	−1.0 to −2.9 vs. +1.8	−1.3 to −8.1 vs. −0.7	NA
**TC change**	−3.2% vs. +0.5%	−4.9% to −7.4% vs. −1.1%	−6.2 to −9.7% vs. −2.5%	−0.1 to +0.1 vs. (i) −0.2 (ii) 0	NA	−0.5 to −0.7 vs. (i) +0.1 (ii) −0.1	−6.2 to −12.2% vs. +1.3%	NA	−11.6% to −15.1% vs. −2.8%	−0.2 to −0.9 vs. +0.1	−0.5 to −0.9 vs. −1.1
**TG change**	−26.9% vs. −4.3%	−24.3% to −31.4% vs. −6.3%	−7.2 to −14.1% vs. +0.8%	−0.2 to −0.3 vs. (i) −0.3 (ii) −0.1	NA	−0.3 to −0.6 vs. (i) −0.1 (ii) −0.1	−26.6 to 36.4% vs. +0.2%	NA	−21.7% to −34.9% vs. +7.3%	−0.3 to −0.6 vs. 0	−0.1 to −0.4 vs. −0.3
**Safety outcomes**											
**Any AE (%)**	92% vs. 86%	79–82% vs. 72%	83–97% vs. 76%	71–88% vs. (i) 81% (ii) 66%	90–92% vs. 75%	90–93%% vs. (i) 81% (ii) 72%	94–97% vs. 81%	100% vs. 88%	NA	73–94% vs. 70%	92–100% vs. 96%
**SAE (%)**	10% vs. 9%	5–7% vs. 7%	0–10% vs. 0%	2–7% vs. (i) 4% (ii) 3%	1–8% vs. 7%	2–5% vs. (i) 3% (ii) 7%	2–7% vs. 0%	0% vs. 0%	0–1% vs. 0%	0–6% vs. 4%	0–8% vs. 0%
**AE leading to medication discontinuation (%)**	6% vs. 4%	4–7% vs. 3%	10–21% vs. 2%	1–6% vs. (i) 7% (ii) 3%	20–29% vs. 4%	20–34% vs. (i) 18% (ii) 3%	0–2% vs. 0%	0% vs. 0%	5–20% vs. 6%	6–16% vs. 8%	0–8% vs. 0%
**Nausea (%)**	52% vs. 15%	25–33% vs. 10%	37–58% vs. 10%	20–47% vs. (i) 39% (ii) 18%	34–65% vs. 20%	51–68% vs. (i) 40% (ii) 7%	21–41% vs. 5%	13–63% vs. 13%	26–60% vs. 11%	14–60% vs. 11%	50–83% vs. 33%
**Vomiting (%)**	24% vs. 4%	8–12% vs. 2%	14–32% vs. 6%	6–8% vs. (i) 20% (ii) 3%	9–35% vs. 5%	20–55% vs. (i) 17% (ii) 0%	13–28% vs. 3%	38% vs. 0%	6–28% vs. 3%	3–26% vs. 1%	0–75% vs. 13%
**Diarrhoea (%)**	27% vs. 17%	19–23% vs. 7%	3–36% vs. 10%	7–18% vs. (i) 18% (ii) 9%	18–28% vs. 10%	14–20% vs. (i) 23% (ii) 5%	19–31% vs. 15%	25–50% vs. 50%	8–19% vs. 5%	9–20% vs. 11%	0–17% vs. 38%
**Constipation (%)**	28% vs. 15%	12–17% vs. 6%	13–32% vs. 6%	8–21% vs. (i) 26% (ii) 7%	12–26% vs. 5%	12–18% vs. (i) 17% (ii) 5%	NA	NA	13–23% vs. 5%	6–16% vs. 3%	NA

*GLP-1* glucagon-like peptide-1, *GIP* glucose-dependent insulinotropic polypeptide, *GCG* glucagon, *PO* oral, *SC* subcutaneous, *OD* once-daily, *OW* once-weekly, *MoA* mechanisms of action, *WL* weight loss, *WC* waist circumferance, *SBP* systolic blood pressure, *DBP* diastolic blood pressure, *TC* total cholesterol, *TG* triglycerides, *ETD* estimated treatment difference, *AE* adverse events, *SAE* serious adverse events, *lira* liraglutide, *sema* semaglutide, *plb* placebo, *NA* not available/not reported. Data presented as mean change unless stated otherwise.

*Data from published abstract, clinicaltrial.gov or from press-release by the manufacturing company; ^ Phase I data

^A^data presented as efficacy estimand, ^B^efficacy estimand data not available, primary analysis is presented ^C^unspecified whether data is efficacy estimand or treatment policy estimand

**Fig. 4:**
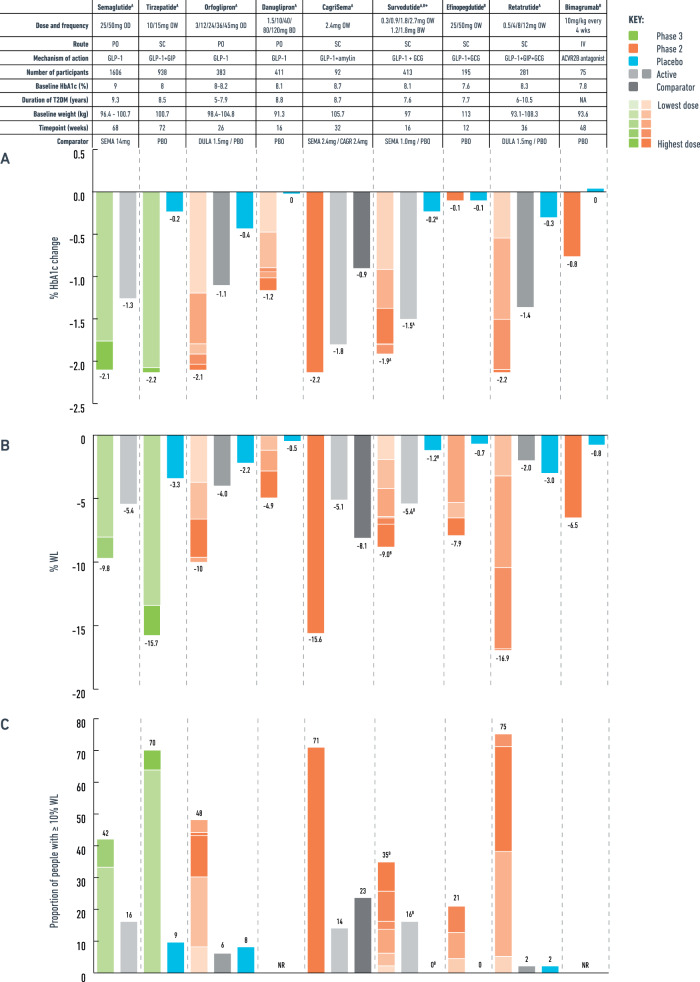
HbA1c reduction and weight loss in people with type 2 diabetes with the pipeline molecules for obesity management. **A** Mean HbA1c change, **B** Mean % weight loss, **C** Proportion of people achieving ≥10% weight loss. OD once daily, OW once weekly, BW twice weekly, PO oral, SC subcutaneous, IV intravenous, T2DM type 2 diabetes, GLP-1 glucagon like peptide-1, GIP glucose-dependent insulinotropic polypeptide, GCG glucagon, ACVR2B activin receptor type 2B, PBO: placebo, SEMA semaglutide, DULA dulaglutide, CAGR cagrilintide, WL weight loss, NA not available, NR not reported. ^A^for efficacy estimand data, ^B^main analysis presented, as efficacy estimand not available, *data from published abstract, presentation, clinicaltrial.gov or from press-release by the manufacturing company.

In people with T2D, oral semaglutide 50 mg once daily resulted in 9.8% WL vs. 5.4% WL with 14 mg oral semaglutide after 68 weeks at the PIONEER PLUS trial (Fig. 4, Supplementary Table 2). Additionally, oral semaglutide 50 mg reduced the HbA1c by −2.1% at 68 weeks compared to −1.3% with oral semaglutide 14 mg (Fig. 4) [22].

### Orforglipron

Orforglipron is a once-daily, oral, non-peptide GLP-1 RA (small molecule) which interacts with the GLP-1 receptor in a slightly different manner compared to native GLP-1. More specifically, orforglipron is a potent partial GLP-1 RA which is biased towards G-protein activation over to β-arrestin recruitment at the GLP-1 receptor [23]. Orforglipron is under assessment for the management of obesity and T2D and may provide a competitive alternative to oral semaglutide, with less burdensome administration, as it does not require to be taken at a fasting state [24].

In people with obesity, 36 weeks of orforglipron (doses ranging from 12 to 45 mg in a phase 2 trial) resulted in a dose-dependent WL up to −14.7% compared to −2.3% with placebo, with concomitant improvement in cardiometabolic risk factors (Table 2) [25].

In people with T2D, 48% of the participants achieved ≥10% WL after 26 weeks of treatment with orforglipron 45 mg in a phase 2 trial (Fig. 4, Table 3). The mean HbA1c change was up to −2.1% with orforglipron 45 mg vs. −0.4% with placebo and −1.1% with dulaglutide [24].

**Table 3:** Efficacy and safety outcomes with the pipeline molecules for obesity treatment in people with type 2 diabetes.

Medication	Oral semaglutide [22] 25, 50 mg^A^	Tirzepatide [47] 10, 15 mg** ^A^	Danuglipron [28] 2.5, 10, 40, 80, 120 mg^A^	Orfoglipron [24] 3,12, 24, 36, 45 mg^A^	CagriSema [89] 2.4/2.4 mg^A^	Survodutide* [69] 0.3, 0.9, 1.2,1.8, 2.7^A,B^	Mazdutide [72] 3.0, 4.5, 6.0 mg^B^	Efinopegdutide [78] 5, 7.4,10 mg^B^	Retatrutide [67] 0.5, 4, 8, 12 mg^A^	Bimagrumab [97] 10 mg/kg^B^	Pemvidutide* [76] 1.2, 1.8, 2.4 mg^A^
**Route and frequency**	PO, OD	SC, OW	PO, BD	PO, OD	SC, OW	SC, OW or (1.2, 1.8) BW	SC, OW	SC, OW	SC, OW	IV, every 4 weeks	SC, OW
**Comparator**	vs. oral Sema 14 mg	vs. plb	vs. plb	vs. (i) Dula 1.5 mg vs. (ii) plb	vs. (i) Sema 2.4 mg vs. (ii) Cagrilintide 2.4 mg	vs. (i) Sema 1 mg vs. (ii) plb	vs. (i) Dula 1.5 mg vs. (ii) plb	vs. plb	vs. (i) Dula 1.5 mg vs. (ii) plb	vs. plb	vs. plb
**MoA**	GLP-1	GLP-1 + GIP	GLP-1	GLP-1	GLP-1 + amylin	GLP-1 + GCG	GLP-1 + GCG	GLP-1 + GCG	GLP-1 + GIP + GCG	ACVR2B antagonist	GLP-1 + GCG
**Trial phase**	3	3	2	2	2	2	2	2	2	2	1
**Trial duration**	68 weeks	72 weeks	16 weeks	26 weeks	32 weeks	16 weeks	20 weeks	12 weeks	36 weeks	48 weeks	12 weeks
**Participants**	1070 vs. 536	1876 vs. 938	258 vs. 57	278 vs. (i) 50 (ii) 55	31 vs. (i) 31 (ii) 30	236 vs. (i) 45 (ii) 49	149 vs. (i) 50 (ii) 51	146 vs. 49	184 vs. (i) 46 (ii) 45	37 vs. 38	40 vs. 14
**Efficacy outcomes**											
**WL%**	−5.4% to −9.8% vs. −5.4%	−13.4% to −15.7% vs. −3.3%	+0% to −4.9% vs. −0.5%**	−3.7% to −10% vs. (i) −4% (ii) −2.2%	−15.6% vs. (i) −5.1% (ii) −8.1%	−1.9% to −9% vs. (i) −5.4% (ii) −1.2%^B^	−4.1% to −7.1% vs. (i) −2.7% (ii) −1.4%	−5.3% to −7.9% vs. −0.7%	−3.2% to −16.9% vs. (i) −2% (ii) −3%	−6.5% vs. −0.8%	−4.4% to −7.7% vs. +0.8%
**≥5% WL**	63–73% vs. 47%	82–86% vs. 31%	6–27% vs. 2%	33–81% vs. (i) 36% (ii) 22%	NA	8–57% vs. (i) 39% (ii) 68%	24–57% vs. (i) 18% (ii) 10%	43-63% vs. 2%	NA	65% vs. 10%	NA
**≥10% WL**	34–42% vs. 16%	63–70% vs. 9%	NA	8–48% vs. (i) 6% (ii) 8%	71% vs. (i) 14% (ii) 23%	2–35% vs. (i) 16% (ii) 0	10–24% vs. (i) 0% (ii) 0%	4–21% vs. 0%	5–75% vs. (i) 2% (ii) 2%	NA	NA
**≥15% WL**	NA	41–52% vs. 3%	NA	4–24% vs. (i) 2% (ii) 2%	54% vs. (i) 0 (ii) 7%	NA	NA	NA	0–63% vs. (i) 0% (ii) 2%	NA	NA
**≥20% WL**	NA	23-34% vs. 1%	NA	NA	NA	NA	NA	NA	0–40% vs. (i) 0% (ii) 2%	NA	NA
**HbA1c (%)**	−1.7 to −2.1 vs. −1.3	−2.1 to −2.2 vs. −0.2	−0.5 to −1.2 vs. 0	−1.2 to 2.1 vs. (i) −1.1 (ii) −0.4	−2.2 vs. (i) −1.8 (ii) −0.9	−1.9 vs. (i) −1.5 (ii) −0.2^A^	−1.4% to 1.7% vs. (i) −1.4% (ii) 0%	−0 to −0.1 vs. −0.1	−0.5 to −2.2 vs. (i) −1.4 (ii) −0.3	−0.8 vs. 0	0 to +0.1 vs. +0.4
**HbA1c ≤6.5%**	42–56% vs. 32%	84–87% vs. 16%	NA	45–84% vs. (i) 41% (ii) 15%	75% vs. (i) 48% (ii) 17%	NA	28–56% vs. (i) 46% (ii) 8%	NA	15–82% vs. (i) 43% (ii) 8%	NA	NA
**HbA1c<7%**	58–70% vs. 50%	90–91% vs. 29%	31–65% vs. 8%	65–96% vs. (i) 64% (ii) 24%	89% vs. (i) 69% (ii) 33%	NA	54–73% vs. (i) 60% (ii) 18%	NA	37–82% vs. (i) 60% (ii) 22%	NA	NA
**WC change (cm)**	−7 to −8 vs. −5	−10.8 to −13.1 vs. −3.3	NA	−2.6 to −8.7 vs. (i) −4.2 (ii) −2.8	NA	−1.8 to −12.9 vs. (i) −3.6 (ii) −2.0	−3.1 to −5.1 vs. (i) −2.6 (ii) −0.4	NA	−2.2 to −13.2 vs. (i) −2.2 (ii) −0.9	−9.0 vs. 0.5	NA
**SBP change (mmHg)**	−5.0 to −6.3 vs. −4.2	−5.9 to −7.7 vs. −1.2	−2.8 to −6.7 vs. −1.7	−6.7 to −8.7 vs. (i) −7.9 (ii) −5.5	−13 vs. (i) +1 (ii) −2	NA	−6.1 to −8.9 vs. (i) −3.5 (ii) −1.3	−1.0 to −7.8 vs. +2.0	−2.8 to −8.8 vs. (i) −1.5 (ii) +1.5	NA	NA
**DBP change (mmHg)**	−2.3 to −2.7 vs. −2.4	−2.1–2.9% vs. 0%	+0.2 to -1.2 vs. -1.3	−1.1 to −2.3 vs. (i) −2.5 (ii) -1.8	−4 vs. (i) 0 (ii) -2	NA	−1.6 to −4.5 vs. (i) −1.9 (ii) −0.8	−2.3 to −6.1 vs. 0	−1.6 to −3.9 vs. (i) +0 (ii) −1.2	NA	NA
**TC change (mmol/l)**	1.0 vs. 1.0^	−2% vs. 3%	NA	−1.2 to −7.3% vs. (i) −1% (ii) +5.6%	0.9 vs. (i) 0.9 (ii) 1^	NA	−0.1 to −0.4 vs. (i) +0.2 (ii) +0.5	−0.3 to −0.4 vs. 0	−7 to −16.7% vs. (i) −0.9% (ii) −2.2%	0 vs. 0	NA
**TG change (mmol/l)**	0.7–0.8 vs. 0.8^	−27% vs. −3%	NA	+1.6 to −16.1% vs. (i) 0.2% (ii) +4.6%	0.7 vs. (i) 0.8 (ii) 0.8^	0.7 vs. (i) 0.8 (ii) 0.8^	−0.2 to −0.8 vs. (i) −0.3 (ii) +0.3	−0.3 to −0.5 vs. −0.1	−9.8 to 35% vs. (i) −4.3% (ii) −9.9%	0 vs. 0	NA
**Safety outcomes**											
**Any AE (%)**	79–80% vs. 76%	71–78% vs. 76%	46–64% vs. 48%	62–89% vs. (i) 56% (ii) 62%	68% vs. (i) 71% (ii) 80%	54–77% vs. (i) 40% (ii) 31%	76–84% vs. (i) 76% (ii) 65%	63–80% vs. 57%	55–79% vs. (i) 67% (ii) 62%	84% vs. 82%	NA
**SAE (%)**	8–11% vs. 10%	6–9% vs. 7%	1–8% vs. 2%	0–11% vs. (i) 2% (ii) 6%	0% vs. (i) 6% (ii) 13%	0–8% vs. (i) 5% (ii) 0%	0–6% vs. (i) 8% (ii) 8%	2–6% vs. 2%	4–8% vs. (i) 2% (ii) 7%	8% vs. 8%	0% vs. 7%
**AE leading to medication discontinuation (%)**	12–13% vs. 10%	4–7% vs. 4%	3–34% vs. 8%	12–19% vs. (i) 4% (ii) 6%	0% vs. (i) 3% (ii) 0%	10–30% vs. (i) 4% (ii) 5%	0% vs. (i) 2% (ii) 0%	10–25% vs. 4%	2–17% vs. (i) 2% (ii) 4%	14% vs. 0%	0% vs. 0%
**Hypoglycaemia (%)**	14–17% vs. 13%	4–5% vs. 1%	1–9% vs. 0%	2–8% vs. (i) 4% (ii) 4%	6% vs. (i) 0% (ii) 7%	0–6% vs. (i) 8% (ii) 3%	6–16% vs. (i) 2% (ii) 8%	NA	0–4% vs. (i) 0% (ii) 0%	NA	NA
**Nausea (%)**	27% vs. 18%	20–22% vs. 6%	7–33% vs. 3%	24–38% vs. (i) 18% (ii) 6%	29% vs. (i) 16% (ii) 13%	20–48% vs. (i) 12% (ii) 8%	22–25% vs. (i) 30% (ii) 6%	27–43% vs. 10%	4–42% vs. (i) 17% (ii) 4%	11% vs. 0%	0–23% vs. 0%
**Vomiting (%)**	17–18% vs. 10%	11–13% vs. 3%	0–25% vs. 0%	6–36% vs. (i) 8% (ii) 2%	10% vs. (i) 3% (ii) 0%	14–26% vs. (i) 4% (ii) 5%	12–16% vs. (i) 14% (ii) 2%	17–35% vs. 0%	0–17% vs. (i) 9% (ii) 2%	NA	0–8% vs. 0%
**Diarrhoea (%)**	13–14% vs. 12%	20–22% vs. 9%	4–18% vs. 3%	6–29% vs. (i) 12% (ii) 7%	16% vs. (i) 6% (ii) 7%	12–26% vs. (i) 10% (ii) 12%	37–40% vs. (i) 16% (ii) 8%	4–12% vs. 4%	2–25% vs. (i) 9% (ii) 4%	41% vs. 11%	0–8% vs. 0%
**Constipation (%)**	6–7% vs. 7%	8–9% vs. 4%	NA	3-22% vs. (i) 0% (ii) 2%	16% vs. (i) 13% (ii) 13%	4–16% vs. (i) 6% (ii) 0%	NA	8–18% vs. 4%	6–17% vs. (i) 7% (ii) 2%	NA	0–15% vs. 7%

*GLP-1* glucagon-like peptide-1, *GIP* glucose-dependent insulinotropic polypeptide, *GCG* glucagon, *PO* oral, *SC* subcutaneous, *OD* once daily, *BD* twice daily, *OW* once-weekly, *BW* twice-weekly, *MoA* mechanisms of action, *WL* weight loss, *WC* waist circumferance, *SBP* systolic blood pressure, *DBP* diastolic blood pressure, *TC* total cholesterol, *TG* triglycerides, *ETD* estimated treatment difference, *AE* adverse events, *SAE* serious adverse events, *dula* dulaglutide, *sema* semaglutide, *plb* placebo, *NA* not available/not reported. Data presented as mean change unless stated otherwise;

*Data from published abstract, clinicaltrial.gov or from press-release by the manufacturing company **obesity trial, ^ Ratio of week 68 to baseline

^A^data presented as efficacy estimand, ^B^efficacy estimand data not available, primary analysis is presented.

### Danuglipron

Danuglipron is another oral, non-peptide, G-protein biased GLP-1 RA [26]. Recently, a phase 2b study for people with obesity has been completed and a press release revealed that danuglipron doses between 40 and 200 mg twice daily led up to 11.7% WL compared to 1.4% weight gain with placebo after 32 weeks of treatment [27]. However, the medication discontinuation rates were greater than 50% across all doses compared to ≈40% with placebo, with the most common adverse events (AE) being gastrointestinal in nature [27].

In people with T2D and overweight/obesity, 16 weeks of danuglipron led to a placebo-adjusted WL up to −4.2 kg with the highest dose of 120 mg twice daily. A mean placebo-adjusted reduction of HbA1c up to −1.2% with the highest dose was also observed [28].

Currently, multiple phase 3 trials with oral semaglutide and orforglipron are ongoing for different populations (NCT05803421), while another oral GLP-1 RA (CT-996) is in early phase clinical trials (Table 1).

### Adverse events with oral GLP-1 RA

Similar to injectable GLP-1 RA, the most common AE with oral GLP-1 RA were gastrointestinal which were mild to moderate in severity (Tables 2 and 3, safety outcomes). In the OASIS-1 study, 13% of participants (people with obesity without diabetes) receiving oral semaglutide 50 mg experienced “altered skin sensation” events compared to 1% with placebo - these events were generally mild to moderate in severity, occurred during dose escalation to the higher doses and resolved without requiring permanent treatment discontinuation [21]. The “altered skin sensation” has not been reported in the PIONEER-PLUS study or with injectable semaglutide 2.4 mg and its aetiology will need to be explored further in future studies.

AE leading to discontinuation of treatment were 6–13% with semaglutide 50 mg once daily in phase 3 trials (vs. 4–10% with placebo) and 10–21% with orforglipron in phase 2 trials (vs. 2–6% with placebo, Tables 2 and 3). The percentages of participants who experienced AE leading to treatment discontinuation as well as of those who experienced gastrointestinal AE with oral semaglutide 50 mg (Tables 2 and 3) were similar to those observed with subcutaneous semaglutide 2.4 mg (in STEP-1 and STEP-2 trials 6–7% discontinued treatment due to AE, 34–44% experienced nausea, 21–32% diarrhoea and 22–25% vomiting) [11, 29].

For orforglipron, participants initiated on higher doses and those on rapid titration schemes appeared to have higher discontinuation rates, providing insights into the optimisation process for the phase 3 trials.

For danuglipron, most AE were mild in severity at the phase 2 trial for people with T2D, but 22–34% of participants receiving the higher doses (≥80 mg twice daily) discontinued the medication due to AE (vs. 8% with placebo, Table 3). As the discontinuation rates were also high with the twice daily danuglipron at the phase 2 obesity trial (as discussed above), twice daily danuglirpon formulation will not advance into phase 3 studies, but an improved once-daily formulation with an aim to improve tolerability profile is currently under assessment [27]. Another oral, non-peptide GLP-1 RA (lotiglipron) has also been withdrawn from development as in early phase clinical trials resulted in elevated liver enzymes which could indicate liver toxicity [30].

## Other entero-pancreatic hormones and combination of entero-pancreatic hormones in the management of obesity

Numerous entero-pancreatic hormones [GIP, glucagon, amylin and peptide YY (PYY) agonists and GIP antagonists] with diverse metabolic actions are currently under investigation either alone or in combination with GLP-1 RA (Fig. 2), aiming to enhance and/or complement the effect of GLP-1 agonism on weight and metabolism [14]. The notion for combining entero-pancreatic hormone-based therapies for obesity treatment is supported by the efficacy of bariatric surgery on WL, an intervention leading to increased postprandial levels of multiple entero-pancreatic hormones [31, 32]. Moreover, preclinical and early phase clinical trials have confirmed the therapeutic potential of entero-pancreatic hormone combinations in obesity and/or metabolic complications such as T2D [33].

## GIP agonism

GIP is secreted by K-cells in jejunum in response to food intake and its physiological role includes stimulation of insulin secretion, increased glucagon secretion, increased lipogenesis and enhancement of lipid buffering capacity (Fig. 1) [34]. In the context of T2D, the ability of GIP to stimulate insulin secretion and to ameliorate glycaemia is impaired [35].

Studies in animal models have shown an anorexigenic action of GIP receptor agonism [36] and a recent phase 1 clinical trial with a long-acting GIP RA found that repeated dosing for 4 weeks induced modest WL (−1.9 to −3.1 kg vs. −0.4 kg with placebo) in people with T2D, without delay in gastric emptying or nausea and vomiting [37].

Nevertheless, as the simultaneous activation of GLP-1 and GIP receptors in preclinical models results in greater WL and glucose-lowering efficacy compared to activation of each receptor alone [38–40], the interest for the development of unimolecular GLP-1 and GIP RA was increased, despite that this additive effect in food intake and glycaemia was not observed in some acute clinical studies [41, 42].

## Dual GLP1 and GIP agonists

Tirzepatide is a once weekly, subcutaneous, unimolecular dual RA (GLP-1 and GIP) which has comparable GIP receptor binding affinity to native GIP and 5 times lower GLP-1 receptor affinity than that of native GLP-1. Tirzepatide has been approved for T2D management - the mean HbA1c reduction in SURPASS programme ranged between 1.9 and 2.6% across the trials and 40–69% of participants achieved ≥10% WL with the higher doses (10 and 15 mg), despite that there was no additional support for lifestyle intervention [16, 43]. In a subanalysis of SURPASS-3, tirzepatide 10 and 15 mg resulted also in a relative reduction in liver fat content by 40–47% compared to 11% with insulin glargine at 52 weeks [44]. A significant reduction in appetite and food intake was also observed with tirzepatide compared to placebo in people with T2D [45].

The phase 3 SURMOUNT programme assessed the safety and efficacy of tirzepatide as obesity treatment and the medication has now been approved (November 2023) for chronic weight management by the US Food and Drug Administration (FDA) and the European Medicines Agency (EMA) [46, 47]. In SURMOUNT-1, 72 weeks of tirzepatide induced mean WL of 16–22.5% in people without diabetes compared to 2.4% with placebo, with no sign of the weight plateauing, suggesting that there may be further WL with long-term use [46]. In SURMOUNT-2, the mean WL was up to 15.7% with tirzepatide 15 mg compared to 3.3% WL with placebo at 72 weeks for people with obesity and T2D [47]. In both studies, improvements in weight associated with improved quality of life parameters, physical function and cardiometabolic risk factors [47].

The most commonly reported AE with tirzepatide were gastrointestinal, including nausea, diarrhoea and vomiting. Most of them were mild to moderate in severity and improved over time. Only 4-7% of participants in SURMOUNT-1 and −2 discontinued the medication due to AE (Tables 2, 3).

Another two trials from the SURMOUNT programme have recently been published [48, 49]. SURMOUNT-3 evaluated the efficacy and safety of tirzepatide compared to placebo for 72 weeks after a 12-week intensive lifestyle intervention lead-in period that included a low-calorie diet and exercise. The trial randomised adults with obesity who have achieved ≥ 5% WL by the end of the 12-week lead-in period (mean WL at the lead-in period was 6.9%) to placebo or tirzepatide. Those taking tirzepatide, achieved an additional 18.4% WL from randomisation to 72 weeks compared to those taking placebo who experienced weight regain of 2.5% [48].

The SURMOUNT-4 trial assessed the weight maintenance with tirzepatide and had also two periods: a 36-week open-label lead-in period during which all participants took tirzepatide, followed by a 52-week double-blind treatment period during which participants were randomised to either continue on tirzepatide or switch to placebo. At the end of the 36-week tirzepatide lead-in period, participants achieved 21.1% mean WL. Those taking tirzepatide, managed an additional 5.5% WL from randomisation, compared to those taking placebo who experienced mean weight regain of 14% from randomisation [49]. At the end of the SURMOUNT-4 study (88 weeks from baseline), 25.9% of people at the placebo group were able to achieve ≥ 15% WL compared to 84.1% at the tirzepatide group.

Several other trials are assessing the safety and efficacy of tirzepatide in improving different cardiometabolic complications such as OSA, MASH and heart failure with preserved ejection fraction (HFpEF) (NCT05412004, NCT04166773, NCT 04847557) [16]. Moreover, multiple other GLP-1/GIP RA in oral or injectable form are in early phase of development (Fig. 1) – CT 388 has shown a placebo-adjusted WL of 8.5% after 4 weeks in a phase 1 trial [50].

The exact molecular mechanisms leading to WL and glycaemia improvements with tirzepatide are still under investigation, mainly because the role of GIP receptor activation with tirzepatide is unclear. One prevailing theory is that tirzepatide is an imbalanced and biased GLP-1 RA with very low efficacy for recruitment of β-arrestin, whilst is acting as an unbiased GIP agonist with full efficacy for β-arrestin recruitment [51, 52]. Nevertheless, a number of other hypotheses have been suggested and both GIP receptor activation and GIP receptor antagonism have been proposed as potential mechanisms to tirzepatide’s efficacy [53].

## GLP-1 agonists and GIP antagonists

GIP antagonism is also a potential obesity treatment as in preclinical studies improved the metabolic profile and reduced food intake [54–56]. A possible explanation for the similar effects on weight of both GIP agonism and antagonism is the potential desensitisation of GIP receptors by GIP agonists exposure [57].

AMG133 (subcutaneous, every 4 weeks) is a monoclonal antibody that was designed to antagonise the GIP receptor and is also linked to two modified GLP-1 peptides that activate the GLP-1 receptor [58]. Results from a phase 1 trial have supported further clinical evaluation with a dose-dependent WL up to 14.5% with AMG133 compared to 1.5% with placebo by day 85 [59]. Of note, the WL in participants to this trial was maintained for few months after the last injection. AE were mainly nausea and vomiting which were transient. A 52-week phase 2 trial on AMG133 in people with overweight and obesity is ongoing (NCT05669599).

## GLP-1 and glucagon co-agonists

Glucagon is secreted from the pancreatic a-cells and the main physiological site of action is the liver to increase hepatic glucose production [60]. Glucagon agonism reduces also food intake [61] and increases energy expenditure [62], suggesting that it could promote WL (Fig. 1). In vivo studies with long-acting glucagon analogue suggest that hypoaminoacidaemia could be an important contributing mechanism to the observed increase in energy expenditure with glucagon agonism, but it also leads to enhanced lean mass loss [63]. Moreover, the combination of glucagon with GLP-1 actions could improve WL while protecting against the risk of hyperglycaemia [64]. Further glucagon actions include the improvement of the whole body lipid metabolism and the promotion of hepatic fatty acid oxidation which may provide therapeutic actions for MASLD and/or MASH [60].

Initial results from studies in rodents suggested a synergistic role of dual GLP-1 and glucagon agonism on reducing food intake and led to the development of numerous unimolecular GLP-1/glucagon co-agonists. Despite the encouraging results of GLP-1/glucagon co-agonism in experimental models, different GLP-1 and glucagon co-agonists have shown various levels of efficacy and tolerability in people with obesity and/or T2D, in early phase clinical trials, which may be explained by the different ratio of GLP-1 to glucagon activity between the different molecules [65].

Additionally, clinical trials with long-acting dual or triple agonists targeting the glucagon receptor have demonstrated a marked suppressive effect on circulating amino-acids in both humans and animals [66, 67]. This could be due to enhancement of hepatic amino-acid catabolism with hepatic glucagon receptor activation. Over the next years, it will be important to understand the effect of the reduced circulating amino-acids with the novel multi-agonists targeting the glucagon receptor on lean muscle mass (especially in populations with obesity and high risk of sarcopenia) as well as on energy expenditure [66].

Survodutide (BI 456906) is a GLP-1/glucagon co-agonist which has progressed to phase 3 clinical trials as treatment for obesity (SYNCHRONIZE programme). In a recently completed phase 2 trial (abstract data), 46 weeks of survodutide once weekly (0.6 to 4.8 mg) resulted in a dose-dependent mean WL up to 18.7% vs. 2% WL with placebo in people with obesity [68]. The percentage of participants who discontinued the medication due to AE ranged between 20 and 29%. Most treatment discontinuations were due to gastrointestinal AE and occurred during the rapid escalation phase – this may be mitigated with more gradual dose escalation at phase 3 trials.

For people with T2D, survodutide 1.8 mg twice weekly resulted in 9% WL vs. 5.4% WL with semaglutide 1 mg and 1.2% WL with placebo at 16 weeks (phase 2 trial) [69]. Mean HbA1c reduction was superior with the higher doses of survodutide compared to semaglutide 1 mg (1.9% vs. 1.5%, respectively). Despite that the serious adverse events (SAE) were similar to semaglutide 1 mg, the AE leading to drug discontinuation were 10–30% compared to 4% with semaglutide 1 mg (Table 3). Survodutide has also received FDA fast-track designation for adults with MASH (NCT04771273).

Mazdutide (IBI362 or LY3305677) is a once-weekly oxyntomodulin analogue which acts both on GLP-1 and glucagon receptors [65]. A phase 2 trial assessing mazdutide 3, 4.5 and 6 mg in a Chinese population with overweight/obesity demonstrates up to 11.3% WL after 24 weeks of treatment compared to 1% weight gain with placebo [70]. Another phase 2 trial using a higher dose of mazdutide for obesity (9 mg) is currently ongoing with a press release revealing a placebo-adjusted WL of 15.4% after 24 weeks [71]. Phase 3 studies using mazdutide as obesity treatment in a Chinese population (doses between 4 and 9 mg) are also ongoing (NCT05607680, NCT06164873).

For people with T2D, a phase 2 trial with mazdutide (doses 3, 4.5 and 6 mg) has shown reduction in HbA1c (up to −1.7%) and up to 7.1% WL at 20 weeks (Table 3), while early phase trials for MASH are in progress [72]. Regarding safety profile, in phase 2 trials for obesity and diabetes, mazdutide was discontinued due to AE by 0–2% of participants and SAE experienced by up to 7% of participants with mazdutide 6 mg (Tables 2 and 3).

Pemvidutide (ALT-801) is another unimolecular once weekly GLP-1/glucagon agonist undergoing a phase 2 trial as obesity treatment, following a phase 1 trial demonstrating up to 10.3% WL at 12 weeks [73]. A press release of the phase 2 obesity trial (MOMENTUM-1) reports a mean WL up to 15.6% (placebo-adjusted 13.4%) at 48 weeks with the 2.4 mg dose. However, 20% of participants discontinued the pemvidutide 2.4 mg due to AE with the most common being nausea and vomiting - the discontinuation rate was 5–19% with lower doses [74]. In people with T2D, a phase 1b study did not demonstrate improvement in HbA1c, however there was a placebo-adjusted WL of 8.5% after 12 weeks (Table 3). Furthermore, in a phase 2 trial in people with MASLD, 24 weeks of pemvidutide resulted in a relative reduction in liver fat content up to 76% with the 2.4 mg dose vs. 14% with placebo [75, 76]. Pemvidutide is also undergoing phase 2 studies as MASH treatment.

Efinopegdutide (JNJ-64565111; HM12525A) is also a unimolecular once weekly GLP-1 and glucagon agonist that has been investigated in people with obesity, T2D and MASLD/MASH. In a phase 2 clinical trial for obesity, 26 weeks of efinopegdutide (doses 5 to 10mg) resulted in up to 11.8% WL in people with obesity (without diabetes) compared to 7.5% WL with liraglutide 3 mg and 1.8% WL with placebo [77, 78]. For people with T2D, a phase 2 trial showed that 12 weeks of efinopegdutide achieve up to 7.9% WL (compared to 0.7% WL with placebo) without actual improvement in HbA1c [78]. However, in people with T2D and MASLD, 24 weeks of efinopegdutide 10 mg reduced liver fat content by 72.7% compared to 42.3% reduction with semaglutide 1 mg despite similar WL achieved with both interventions [79]. Efinopegdutide has been granted fast track designation by the FDA for the treatment of MASH (NCT04944992, NCT05877547) and this is the indication that the clinical development programme of this molecule will focus. Most common side effects were gastrointestinal, with 24.5% of participants in the phase 2 obesity trial discontinuing efinopegdutide 10 mg due to AE – however when the same dose was used with gradual escalation at the MASLD trial (starting dose 2.4 mg for 4 weeks, then 5 mg for 4 weeks and then 10 mg), only 5.6% of participants discontinued the medication due to AE.

Other GLP-1 and glucagon co-agonists in early phase clinical trials as treatments for obesity include AZD9550 and LY3305677 (NCT05623839).

## Triple agonists with GLP1, GIP and glucagon

Given the efficacy and benefits of the dual GLP-1/GIP RA tirzepatide and the dual GLP-1/glucagon RA, a triple agonist targeting all three receptors (GLP-1/GIP/glucagon) may result in superior WL and glycaemic control than dual agonists. Indeed, in pre-clinical models, retatrutide, a triple agonist (GLP-1/GIP/glucagon), resulted in greater WL and improved glucose profile compared to tirzepatide through increased energy expenditure and reduced calorie intake [80, 81].

Retatrutide is administered once weekly and is more potent at human GIP receptors and less potent at GLP-1 and glucagon receptors [80]. In a phase 2 study in people with obesity (without T2D), retatrutide (doses 1 to 12 mg) has led to a dose-dependent WL up to 24.2% at the end of the 48-week treatment period compared to 2.1% with placebo [81]. At 48 weeks, WL of ≥25% had occurred in 36–48% of people with the 8 mg and 12 mg doses compared to no one who received placebo. Greater % WL was attained with retatrutide among participants with BMI≥35 kg/m^2^ and among female participants. Marked improvements in lipid profile and blood pressure were also observed compared to placebo [81].

In people with T2D, retatrutide (0.5 to 12 mg) led to substantial reductions in bodyweight and HbA1c compared to both placebo and dulaglutide 1.5 mg in a phase 2 trial [67]. After 36 weeks, retatrutide resulted in HbA1c reduction up to –2.2% vs. −1.4% with dulaglutide and −0.3% with placebo and a bodyweight reduction of up to 16.9% with retatrutide vs. 2% with dulaglutide and 3% with placebo [67]. The benefits of triple agonism extended also to MASLD – in a subgroup of 98 people, fat content normalised in approximately 90% of people receiving the highest retatrutide doses [82].

The safety profile of retatrutide was consistent with that of other incretin-based therapies. Transient and mostly mild-to-moderate gastrointestinal symptoms were the most frequently reported AE and occurred more frequently with the 4 mg starting dose groups rather than the 2 mg starting dose groups [67]. AE leading to drug discontinuation were observed in 16% of participants with the 12 mg dose, but the overall incidence of SAE was low (up to 6-8%).

A programme of phase 3 trials with retatrutide (TRIUMPH) is ongoing with an aim to assess the safety and efficacy of retatrutide in different populations living with obesity (OSA, T2D, established cardiovascular disease, osteoarthritis). Another triple agonist (HM15211) is undergoing phase 1 clinical trials with no published data so far.

## Amylin agonism

Amylin is co-secreted with insulin from the β-cells of the pancreas and plays a key role in postprandial satiety regulation (Fig. 1). Amylin acts on amylin receptors in the brainstem to reduce food intake [83] and improves glucose metabolism by delaying gastric emptying and inhibiting glucagon secretion [84, 85]. The reduction of food intake with amylin is not accompanied by the expected concomitant decrease in energy expenditure through actions at the sympathetic nervous system.

Pramlintide is the first synthetic amylin analogue that approved for diabetes management with WL up to 7.9% [86]. Cagrilintide is a newer long-acting amylin analogue - in a phase 2 trial, once weekly cagrilintide resulted in a dose-dependent WL between 6 and 10.8% compared to 9% with liraglutide 3 mg and 3% with placebo [87].

Other amylin-based molecules which are in early stage clinical trials include long-acting amylin agonists and a dual amylin and calcitonin RA (Table 1).

## Dual agonism with GLP-1 and amylin

As WL with GLP-1 RA and amylin analogues results both from distinct and overlapping pathways, a combination of these entero-pancreatic hormones may induce a synergistic WL effect [84].

In a phase 1b trial, 20 weeks of cagrilintide 2.4 mg once weekly in combination with semaglutide 2.4 mg once weekly (cagrisema) resulted in up to 17.1% WL compared to 9.5% WL with semaglutide 2.4 mg plus placebo in people with obesity [88]. In a phase 2 trial, people with T2D and overweight/obesity achieved greater mean WL with cagrisema 2.4 mg compared to cagrilintide 2.4 mg or semaglutide 2.4 mg alone after 32 weeks of treatment (15.6% WL vs. 8.1% WL vs. 5.1% WL, respectively) [89]. Mean HbA1c reduction with cagrisema was also greater compared to semaglutide 2.4 mg and cagrilintide 2.4 mg alone (−2.2% vs. −1.8% vs. −0.9% respectively).

Gastrointestinal AE with cagrisema were more than with semagutide or cagrilintide monotherapy, however the SAE and AE leading to medication discontinuation were minimal and similar between the groups [87, 90]

A programme of phase 3 clinical trials (REDEFINE) assessing the safety and efficacy of cagrisema in people with obesity (NCT05567796, NCT05394519, NCT05813925) is currently ongoing. Moreover, an oral GLP-1 and amylin co-agonist (amycretin) is also in early phase clinical trials (Table 1).

## Peptide YY (PYY)

PYY is co-secreted from the intestinal L cells together with GLP-1 following food intake. Following secretion, PYY is rapidly cleaved by dipeptidyl peptidase-4 (DPP-4) to its active form (PYY 3-36) acting on neuropeptide Y receptor type 2 (Y2R). Y2R receptor is present in the brain and its agonism results in a reduction in food intake and increased satiety [91]. Studies with PYY agonists administered intravenously have shown reduction in food intake with increased satiety [92, 93], however, a nasal PYY agonist showed minimal efficacy with poor tolerability [94]. Long-acting, subcutaneously administered, PYY RA are undergoing early phase clinical trials as obesity treatments either alone or in combination with GLP-1 RA (Table 1, NCT02568306 and NCT03574584).

In a phase 1 trial, a PYY analogue (Y14 peptide) administered subcutaneously at 7- to 14-days intervals was safe and potentially efficacious (WL between 2.9 and 3.6 kg at 31 days, with a 38–55% reduction in food intake vs. placebo) [95]. NNC0165-1875 has recently completed a phase 2 trial in combination with semaglutide 2.4 mg once weekly (NCT04969939), but results are not currently available.

## Other pharmacotherapies not based on entero-pancreatic hormones

Several therapies that are not based on entero-pancreatic hormones are currently under evaluation for obesity and they represent further therapeutic options, considering their distinct mechanism of WL to the entero-pancreatic hormone therapies.

### Bimagrumab

Bimagrumab is a human monoclonal antibody that stimulates skeletal muscle growth by blocking the activin type II receptor (ActRII) and it is administered as a four-weekly intravenous infusion [96]. In a 48-week phase 2 trial in people with T2D and obesity, 10 mg/kg bimagrumab was associated with a marked reduction of fat mass (20.5% vs. 0.5% in placebo) and increased lean mass (3.6% vs. −0.8% placebo), with total WL of −6.5% compared to −0.8% with placebo [97]. There was also a placebo-adjusted improvement in HbA1c of 0.8% with bimagrumab.

AE were similar between bimagrumab and placebo, although bimagrumab was associated with transiently elevated pancreatic and liver enzymes [97]. Bimagrumab has the potential of improving the WL quality by preserving lean mass and could become an attractive treatment option for sarcopenic obesity. A phase 2 trial assessing different doses of bimagrumab (up to 30 mg/kg) in combination with semaglutide as obesity treatment is ongoing (NCT05616013).

### Growth/differentiation factor-15 (GDF-15)

Another potential therapeutic pathway for obesity pharmacotherapies is through the stress-induced cytokine GDF-15, which is expressed in multiple cell types including cardiomyocytes, adipocytes and macrophages [98]. The GDF-15 RA use as a potential treatment for obesity stems from observations that elevated tumour-secreted GDF-15 is correlated with WL [99]. In mice, GDF-15 increases satiety and reduces food intake through actions in the central nervous system [99]. LY3463251 is the first GDF-15 agonist that has completed a phase 1 clinical trial – WL was 3% after a 12-week treatment period [100]. Several GDF-15 agonists are undergoing early phase clinical trials including NNC0247-0829 and JNJ-9090/CIN-109.

## Potential clinical implications and challenges with the new obesity pharmacotherapies

### A new era in obesity management

Tirzepatide has received FDA and EMA approval for chronic weight management and results in ≥20% mean WL in people without T2D. Multiple other molecules (including combinations of entero-pancreatic hormones and oral GLP-1 RA) are in late phase clinical trials as potential obesity treatments, leading to WL that approaches the efficacy of bariatric surgery [101]. Overall, a new era in obesity care is starting, as multiple effective obesity pharmacotherapies with different mechanisms of action and routes of administration may become available over next years. However, a number of challenges will need to be addressed over next years including a better understanding of the full potential of benefits and risks with the new obesity pharmacotherapies and ensuring wide and equal access for people living with obesity.

### Potential implications of the new pharmacotherapies on obesity management

The mean WL in clinical trials with the most efficacious new obesity pharmacotherapies ranges between 15% and 25% at 1 year (and it may be even higher for some agents that have not reached weight plateau at study completion). The efficacy of the new pharmacotherapies will allow clinicians to treat obesity towards individualised treatment targets, similar to what is happening with other chronic diseases such as T2D or dyslipidaemia. Moreover, the availability of multiple effective obesity pharmacotherapies with different mechanisms of action will provide clinicians the opportunity to select treatments based on patient preference, underlying comorbidities, medication safety profile and treatment response, with an aim to achieve the individualised WL targets, improve the overall health and/or the quality of life [7].

However, as with any WL intervention, there will be heterogeneity in treatment responses, even with the new molecules for obesity treatment [15]. Around 10–30% of participants (especially people with T2D) achieved <10% WL in clinical trials even with the higher doses of the new pharmacotherapies [101, 102]. Moreover, the percentage of participants in clinical trials who stopped the treatment due to AE was 5–15% with most of the new agents, and this percentage was up to 20–30% with some of GLP-1/glucagon RA in phase 2 trials – so a considerable proportion of people may not be able to tolerate the new obesity pharmacotherapies or may be unable to titrate them to the higher and most effective doses. The multiple pharmacological treatment options will enhance clinicians’ ability to identify effective personalised regimens by trying a second medication or combining medications with synergistic or complimentary actions or even escalating treatment to bariatric surgery based on patient preference and underlying comorbidities [7].

As obesity is a complex, chronic and progressive disease, it requires also an individualised and adaptive over time approach [14, 102]. Even people who achieve the treatment goals with the new obesity pharmacotherapies might decide to undergo bariatric surgery to support long-term weight maintenance because either they experience weight regain on the medication or they do not have long-term access to obesity pharmacotherapy, or they prefer not to take obesity pharmacotherapy lifelong. Similar to other chronic diseases, if the treatment for the disease of obesity is stopped, weight regain will likely occur and the health benefits will be diminished [14].

Even for people who have undergone bariatric surgery, inadequate WL and/or clinically important weight regain is common and further support with pharmacotherapy may be required to optimise the surgical outcomes. A multimodal approach that combines surgical and medical approaches towards improving health through the selection of the appropriate treatment and achievement of individualised WL targets should be the standard of care in obesity [14]. The efficacy and safety of the new pharmacotherapies after bariatric surgery require further investigation, however the benefits of GLP-1 RA after bariatric surgery on WL and glycaemia have been shown in clinical trials [103, 104].

A healthy lifestyle is the basis to optimise health outcomes regardless of WL – increased levels of physical activity have multiple health benefits including improvement of body composition, physical function and cardiorespiratory fitness, and a balanced Mediterranean diet may be associated with cardiovascular benefits [105]. As the new obesity pharmacotherapies will lead up to 15–25% mean WL, intensive lifestyle interventions may provide small additional WL benefit to these treatments [11, 106]. However, the risk of cholelithiasis, micronutrient deficiencies and lean muscle mass loss during the rapid WL phase are of particular concern with the new obesity treatments. Adequate nutrition, with focus on protein intake as well as resistance exercise during the WL phase may help people preserve their lean muscle mass and prevent micronutrient deficiencies.

Overall, the introduction of the new obesity pharmacotherapies may shift the focus of the multidisciplinary team on nutrition support during the rapid WL phase and on behavioural changes aiming to support long-term weight maintenance, similar to what is the focus after bariatric surgery. It should be noted that the rate of WL with the novel pharmacotherapies could be individualised, something that is not feasible with bariatric surgery, however the optimal WL rate to optimise the risks for nutritional complications and/or cholelithiasis will need further research [107].

### Towards tailored obesity treatment choices based on obesity-related complications

Ectopic fat deposition in different organs results over time in progressive metabolic dysfunction and the development of organ-specific metabolic complications including T2D, MASLD/MASH and HFpEF [108]. The direct and weight-independent actions of some new obesity pharmacotherapies in ectopic fat of specific organs may further enhance the potential for tailored treatment choices based on patient’s complications. For example, combinations of GLP-1/glucagon RA achieve more liver fat content reduction in people with MASLD compared to GLP-1 RA alone despite similar WL, likely due to the direct effect of glucagon on hepatic lipid oxidation [79]. On the other hand, GLP-1 RA can reduce epicardial fat and semaglutide 2.4 mg have shown to improve physical function and symptoms in people with HFpEF [93]. Understanding the full potential of each molecule in improving both metabolic and mechanical obesity-related complications through research will support personalised pharmacotherapy choices.

### Cardiovascular outcomes, long-term efficacy and safety

In people with T2D and established cardiovascular disease, the international guidelines for T2D recommend the use of GLP-1 RA or sodium glucose co-transporters 2 inhibitors (SGLT-2i) as first-line treatment (independent of HbA1c), based on multiple trials demonstrating their cardio-renal benefits [109]. In people with obesity and established cardiovascular disease but without diabetes, semaglutide 2.4 mg resulted in 20% reduction in major adverse cardiovascular events [(MACE), death from cardiovascular causes, nonfatal myocardial infarction or nonfatal stroke] compared to placebo (SELECT trial) after a mean follow-up of 39.8 months – this is the first trial demonstrating cardiovascular benefit for an obesity pharmacotherapy [110].

Despite the cardioprotective effects of semaglutide 2.4mg, whether the combination of GLP-1 RA with other entero-pancreatic hormones will also improve cardiovascular outcomes in people with obesity and/or T2D needs to be established. The SURPASS-CVOT trial (NCT04255433) will assess the cardiovascular safety of tirzepatide compared to dulaglutide in people with T2D and established atherosclerotic disease [111]. Moreover, the SURMOUNT–MMO (tirzepatide, NCT05556512) will assess the impact of the dual GLP-1/GIP RA in cardiovascular outcomes and all-cause mortality for people with obesity (without diabetes) when the REDEFINE-3 study will assess the impact of cagrisema in people with obesity (with and without T2DM) and established cardiovascular disease. Similarly, the oral GLP-1 RA will also need to demonstrate their cardiovascular safety in these populations (NCT05803421).

Until recently, the published studies with the new obesity pharmacotherapies have had up to 2 years follow-up [12]. The SELECT trial is the first study providing long-term data on the safety and efficacy of semaglutide 2.4 mg [110]. People were able to maintain approximately 10% WL over a 4-year period, and the proportion of participants experiencing SAE during the trial was lower in those assigned to semaglutide 2.4 mg compared to placebo (33.4% vs. 36.4%). A higher proportion of participants experienced gallbladder-related disorders with semaglutide 2.4 mg compared to placebo (2.8% vs. 2.3%) and there was no difference in the percentage of participants with acute pancreatitis or malignant neoplasms between the two groups [110].

The cardiovascular outcome trials for the new obesity pharmacotherapies will help us understand better the long-term benefits and risks with each molecule. As the WL achieved with the novel obesity pharmacotherapies approaches that of bariatric surgery, we will need to assess whether long-term complications observed after bariatric surgery such as increased risk of osteoporosis, fractures, macro- and micronutrient deficiencies as well as self-harm behaviours will also be present with pharmacotherapy [112, 113].

### Equal and long-term access to treatment and cost-effectiveness

One of the most challenging aspects of this new era in obesity management will be the long-term and equal access to the new obesity pharmacotherapies through funding from national health systems and/or private health insurances as there is lack of robust cost-effectiveness data. As older molecules will lose patent over next years and a plethora of new molecules will come to the market, it is likely that competition will have a positive impact on the obesity medication prices [14]. However, research is also needed on the efficacy and cost-effectiveness of different strategies to facilitate long-term WL maintenance – for example the use of lower medication doses or the less frequent dosing of obesity pharmacotherapy for WL maintenance.

Moreover, in many national health systems, obesity pharmacotherapy is prescribed in specialist obesity services [114]. The availability of multiple effective obesity pharmacotherapies will increase further the workload in these services and over the next years they will need to be supported with extra personnel. However, similar to other chronic diseases such as T2D, the focus in obesity management (including pharmacotherapy prescription) over time will need to be shifted to primary care and collaborative integrated care models to be developed between specialist obesity services and primary care striving to meet the needs of people living with obesity [115]. This process will take time and will require resources for primary care training and the development of sustainable pathways.

## Conclusion

A new era for obesity treatment has commenced where pharmacotherapy with combinations of entero-pancreatic hormones approach the WL efficacy of bariatric surgery. Tirzepatide is the first dual agonist which has been approved for chronic weight management, but numerous other dual and/or triple agonists (cagrisema, retatrutide, mazdutide and survodutide) are also in phase 3 trials as potential treatments for obesity and its metabolic complications. Moreover, oral GLP-1 RA are also under development and will provide an alternative option.

The plethora of efficacious obesity pharmacotherapies with different mechanisms of action will allow tailored treatment plans based on individual’s preference, comorbidities and treatment response. A percentage of people will not be able to tolerate the new pharmacotherapies or achieve the individualised goals and others may not have long-term access to these treatments. Combining different treatment modalities (including lifestyle interventions, pharmacotherapies and bariatric surgery) may support people to achieve individualised long-term goals, maximise health benefits and improve quality of life.

Obesity pharmacotherapy is a rapidly moving field and further research on long-term clinical efficacy, safety and cost-effectiveness will inform better their place in the treatment algorithms for obesity and obesity-related complications over the next years.

## Supplementary information

Supplementary material

## Data Availability

All data generated or analysed during this study are included in this published article and its supplementary information files.
