# Supplementary material for: What is the pipeline for future medications for obesity?
Source: Int J Obes (Lond). 2024 Feb 1;49(3):433–51. doi: 10.1038/s41366-024-01473-y (PMC11971045; doi:10.1038/s41366-024-01473-y)
Supplement: Supplementary file 1 — Supplementary material [file 41366_2024_1473_MOESM1_ESM.docx]

**Supplementary table 1. Baseline characteristics and summary of trial designs of the pipeline for obesity medications for people without T2D**

| **Medication** | **Comparator** | **No. of participants** | **Add on lifestyle intervention** | **Duration of trial; randomisation ratio** | **Primary outcome** | **Baseline characteristics** |
| --- | --- | --- | --- | --- | --- | --- |
| **Obesity Phase 3 trials** | | | | | | |
| Oral Semaglutide  50mg | Placebo | 667 | 500 kcal/day deficit diet + advise for 150 mins/week physical activity | 68 weeks;  1:1 | (1) % change in body weight at week 68 (2) weight reduction of ≥5% at week 68 | Age: 50 yr  Female: 73% % White: 74%  Baseline weight: 105.4kg Baseline BMI: 37.5kg/m^2^ |
| Tirzepatide 5, 10 , 15mg | Placebo | 2539 | 500 kcal/day deficit diet + advise for 150 mins/week physical activity | 72 weeks; 1:1:1:1 | (1) % change in body weight at week 72 (2) weight reduction of ≥5% at week 72 | Age: 44.9 yr Female: 67.5%  % White: 71% Baseline weight: 104.8kg Baseline BMI: 38.0kg/m^2^ |
| **Obesity Phase 2 trials** | | | | | | |
| Orforglipron  12, 24, 36, 45mg | Placebo. | 272 | Healthy eating and exercise education | 36 weeks; 5:5:3:3:3:3:5 | % change in body weight at week 26 | Age: 54.2 yr  Female: 59%  % White: 87-100% Baseline weight: 108.7kg Baseline BMI: 37.9kg/m^2^ |
| Cagrilintide  0.3, 0.6, 1.2, 2.4, 4.5mg | vs (i) Liraglutide 3mg  vs (ii) placebo | 706 | 500 kcal/day deficit diet + advise for 150 mins/week physical activity | 26 weeks; 1:1:1:1:1:1 | % change in body weight at week 26. | Age: 52.3 yr  Female: 62% % White: 77%  Baseline weight: 107.4kg Baseline BMI: 37.8kg/m^2^ |
| Survodutide 0.6, 2.4, 3.6, 4.8mg* | Placebo | 387 | Not reported at the abstract | 46 weeks; 1:1:1:1:1 | % change in body weight at week 46 | Age: 49.1 yr Baseline weight: 105.7kg Baseline BMI: 37.1kg/m^2^ |
| Efinopegdutide  7.4, 10mg | vs (i) Liraglutide 3mg  vs (ii) placebo | 474 | 600 kcal/day deficit diet | 26 weeks; 1:1:2:2:2 | % change in body weight at week 26. | Age: 46.3 yr  Female: 75.1% % White: 89%  Baseline weight: 113.3kg Baseline BMI: 40.5kg/m^2^ |
| Mazdutide  3, 4.5, 6mg | Placebo | 248 | None | 24 weeks;  NA | % change in body weight change at week 24 | Age: 33.6-37.2 yr Baseline weight: 88.5-90.2kg Baseline BMI: 31.7-32kg/m^2^ |
| Mazdutide  9mg* | Placebo | 80 | Not reported at the press release | 24 weeks;  3:1 | % change in body weight change at week 48 | Age: 34yr Baseline weight: 96.9kg Baseline BMI: 34.3kg/m^2^ |
| Pemvidutide  1.2, 1.8, 2.4mg* | Placebo | 391 | Not reported at the abstract | 48 weeks; 1:1:1:1 | % change in body weight at week 48 | Age: 50yr  Female: 75% Baseline weight: 104kg Baseline BMI: 37kg/m^2^ |
| Retatrutide  1, 4, 8, 12mg | Placebo | 338 | Healthy eating and exercise education | 48 weeks; 2:1:1:1:1:2:2 | % change in body weight at 24 weeks | Age: 48.2 yr  Female: 48%  % White: 88%  Baseline weight: 107.7kg Baseline BMI: 37.3kg/m^2^ |
| **Obesity Phase 1 trials** | | | | | | |
| CagriSema  (Cagrilintide 0.16, 0.3, 1.2, 2.4, 4.5mg/Semaglutide 2.4mg) | Placebo | 95 | None | 25 weeks;  3:1 | Number of treatment-emergent adverse events from baseline to the end of the follow-up period | Age: 40.6 yr  Female: 41% % White: 43%  Baseline weight: 95.7kg Baseline BMI: 32.1kg/m^2^ |
| AMG 133* | Placebo | 49 | Not reported at the abstract | NR | Safety | Age: 48 yr Baseline weight: 99.5kg Baseline BMI: 33.4kg/m^2^ |
| **T2D: Type 2 diabetes; HbA1c = glycosylated haemoglobin; BMI = body mass index; SGLT2i = sodium dependent glucose cotransporter 2 inhibitor; GLP-1RA = glucagon-like peptide receptor agonist; DPP-4i = Dipeptidyl Peptidase IV inhibitor; NA = data not avaiable** | | | | | | |
| *** Data from published abstract, clinicaltrial.gov or from press release by the manufacturing company** | | | | |  |  |

**Supplementary Table 2. Baseline characteristics and summary of trial designs of the pipeline molecules for obesity treatment in people with T2D**

| **Medication** | **Comparator** | **No. of participants** | **Add on to** | **Duration of trial; randomisation ratio** | **Primary outcome** | **Baseline characteristics** |
| --- | --- | --- | --- | --- | --- | --- |
| **T2D Phase 3 trials** | | | | | | |
| Oral Semaglutide 25, 50mg | Oral semaglutide 14mg | 1606 | Background treatment: Any oral glycemic-lowering agent except DPP-4i or GLP-1RA | 68 weeks;  1:1:1 | change in HbA1c from baseline to week 52 | Age: 57.6-58.8 yr  Female: 39-43%  % White: 74-81%  Baseline HbA1c: 9%  Diabetes duration: 8.9-9.7 yr  Baseline weight: 96.1-96.6kg  Baseline BMI: 33.7-34.1kg/m^2^ |
| Tirzepatide  10, 15mg** | Placebo | 938 | **Lifestyle intervention:** 500 kcal/day deficit diet + advise for 150 mins/week physical activity  Background treatment: any oral glycemic-lowering agent except DPP-4i or GLP-1RA | 72 weeks;  1:1:1 | (1) % change in body weight at week 72. (2) weight reduction of ≥5% at week 72 | Age: 54.2 yr  Female: 51%  % White: 76%  Baseline weight: 100.7kg  Baseline BMI: 36.1kg/m^2^ |
| **T2D Phase 2 trials** | | | | | | |
| Danuglipron  2.5, 10, 40, 80, 120mg | Placebo | 411 | Background treatment: with or without metformin | 16 weeks; 1:1:1:1:1:1 | change in HbA1c from baseline to week 16 | Age: 58.6 yr  Female: 49%  % White: 83%  Baseline HbA1c: 8.1%  Diabetes duration: 8.8 yr Baseline weight: 91.3kg  Baseline BMI: 32.8kg/m^2^ |
| Orfoglipron  3, 12, 24, 36, 45mg | vs (i) Dulaglutide 1.5mg  vs (ii) placebo | 383 | Background treatment: with or without metformin and/or diet and exercise. | 26 weeks; 5:5:5:5:5:3:3:3:3 | change in HbA1c from baseline to week 26 | Age: 57.4-60.5 yr  Female: 41%  % White: 88-95%  Baseline HbA1c: 8-8.2%  Diabetes duration: 5-7.9 yr Baseline weight: 98.5-104.6kg Baseline BMI: 34.1-36.4kg/m^2^ |
| CagriSema  2.4mg | vs (i) Semaglutide 2.4mg  vs (ii) Cagrilintide 2.4mg | 92 | Background treatment: Metformin ± SGLT2i. | 32 weeks;  1:1:1 | the effect of CagriSema versus semaglutide on HbA1c change from baseline to week 32 | Age: 58 yr  Female: 36%  % White: 78%  Baseline HbA1c: 8.7%  Diabetes duration: 8.7 yr Baseline weight: 105.7kg  Baseline BMI: 35.5kg/m^2^ |
| Survodutide  0.3, 0.9, 1.2, 1.8, 2.7mg* | Placebo | 413 | NA | 17 weeks 1:1:1:1:1 | change in HbA1c from baseline to week 16 | Age: 57.3 yr  Female: 43.3%  Baseline HbA1c: 8.1%  Baseline weight: 97.0kg |
| Mazdutide  3.0, 4.5, 6.0mg | vs (i) Dulaglutide 1.5mg  vs (ii) placebo | 250 | Background treatment: with or without metformin and/or diet and exercise. | 20 weeks  1:1:1:1:1 | Change in HbA1c from baseline to week 20 | Age: 52.5-55.1 yr  Female: 26.5-49%  % Asian: 100%  Baseline HbA1c: 7.9-8.1%  Diabetes duration: 3.3-4.4 yr  Baseline weight: 72.3-78.1kg Baseline BMI: 26.7-28.0kg/m^2^ |
| Efinopegdutide  5, 7.4 and 10mg** | Placebo | 195 | **Lifestyle intervention:** 600 kcal/day deficit diet + advise for physical activity  Background treatment: metformin or up to 2 oral antihyperglycemic agents. | 12 weeks; 1:1:1:1 | % change in bodyweight at week 12 | Age: 56.6 yr  Female: 60.5% % White: 71.3%  Baseline HbA1c: 7.6%  Diabetes duration: 7.7 yr Baseline weight: 113kg  Baseline BMI: 40.3kg/m^2^ |
| Retatrutide  0.5, 4, 8, 12mg | vs (i) Dulaglutide 1.5mg  vs (ii) placebo | 281 | Background treatment: With or without metformin | 36 weeks; 2:2:1:1:1:1:2:2 | change in HbA1c from baseline to week 24 | Age: 56.2 yr  Female: 56%  % White: 84%  Baseline HbA1c: 8.3%  Diabetes duration: 8.1 yr Baseline weight: 98.2kg  Baseline BMI: 35kg/m^2^ |
| Bimagrumab every 4 weeks  (10mg/kg up to a max of 1200mg)** | Placebo | 75 | Background treatment: None or metformin and/or DPP4i | 48 weeks;  1:1 | change in total body fat mass from baseline to week 48 | Age: 60.4 yr  Female: 47%  % White: 76%  Baseline HbA1c: 7.82% Baseline weight: 93.6g  Baseline BMI: 32.9kg/m^2^ |
| **T2D Phase 1 trials** | | | | | | |
| Pemvidutide 1.2, 1.8, 2.4mg* | Placebo | 54 | None | 12 weeks; 1:1:1:1 | NA | NA |
| **T2D: Type 2 diabetes; HbA1c = glycosylated haemoglobin; BMI = body mass index; SGLT2i = sodium dependent glucose cotransporter 2 inhibitor; GLP-1RA = glucagon-like peptide receptor agonist; DPP-4i = Dipeptidyl Peptidase IV inhibitor; NA = data not avaiable** | | | | | | |
| *** Data from published abstract, clinicaltrial.gov or from press release by the manufacturing company **obesity trial** | | | | | |  |
